# AI-Powered Ambient Scribe Technology Experiences Among Emergency Physicians: Cross-Sectional, Mixed Methods Pilot Survey Study

**DOI:** 10.2196/80401

**Published:** 2026-03-03

**Authors:** Taylor Marquis, Matthew Kopp, Jared S Anderson, Anthony M Napoli, Linda L Brown, Yosef Berlyand

**Affiliations:** 1 Department of Emergency Medicine Alpert Medical School Brown University Providence, RI United States

**Keywords:** artificial intelligence, AI, natural language processing, electronic health records, clinical documentation, medical scribes, emergency department operations, health care technology

## Abstract

**Background:**

Health care organizations have started to implement artificial intelligence–powered ambient scribe technology in clinical documentation workflows. Early outpatient studies have shown mixed results. Few studies have evaluated ambient scribes in the emergency department (ED). Due to differences in setting and patient acuity between the ED and ambulatory clinics, there remains a pressing need to research this technology in the ED.

**Objective:**

This study aimed to evaluate emergency physicians’ (EPs) satisfaction, perceived documentation efficiency, after-shift documentation time, and trust in ambient scribe–generated notes compared with in-person scribes or independent documentation, and to identify ED-specific challenges.

**Methods:**

A cross-sectional survey was conducted among 16 board-certified adult and pediatric EPs who were granted access to the ambient scribe technology across 4 EDs. EPs used the ambient scribe for several months before completing a survey with multiple-choice and free-text responses. We performed a mixed methods analysis by summarizing quantitative data through descriptive statistics and performing a practical thematic analysis on free-text responses.

**Results:**

Of the 16 EPs, 14 (87.5%) completed the survey. Among respondents, 9 (64.3%; 95% CI 38.7%-83.7%) reported being satisfied or very satisfied with the ambient scribe, while 3 (21.4%; 95% CI 7.6%-47.6%) expressed dissatisfaction. When given the option, 7 (50%; 95% CI 26.8%-73.2%) respondents preferred the ambient scribe, 4 (28.5%; 95% CI 11.7%-54.6%) preferred in-person scribes, and none (95% CI 0%-20.6%) preferred independent documentation. Among previous users of in-person scribes, 50% (4/8; 95% CI 21.5%-78.5%) favored the ambient scribe. The ambient scribe was reported to improve documentation efficiency (10/14, 71.4%; 95% CI 45.4%-88.3%) and reduce after-shift documentation time (9/14, 64.3%; 95% CI 38.7%-83.7%). However, only 42.9% (6/14; 95% CI 22.3%-69.2%) of respondents trusted the accuracy of ambient scribe–generated notes, compared with 75% (6/8; 95% CI 40.9%-92.9%) who trusted in-person scribes. Few respondents found the ambient scribe helpful for physical examinations (3/13, 23.1%; 95% CI 8.2%-50.3%) or medical decision-making documentation (5/14, 35.7%; 95% CI 16.3%-61.2%). A thematic analysis identified 5 themes: challenges due to the workplace environment, challenges due to the patient population, workflow improvement, workflow harm, and narrow usefulness.

**Conclusions:**

This mixed methods pilot study is among the first to evaluate ambient scribe technology in the ED. Our results add ED-specific insights to literature focused on the outpatient setting. Our findings reveal the potential for enhancing documentation efficiency and reducing administrative burden while highlighting setting-specific challenges. While most EPs preferred artificial intelligence–assisted documentation over independent charting, confidence in documentation accuracy and functionality remains limited compared with human scribes and varies by note component. As the demand for efficiency in emergency medicine continues to grow, scalable solutions such as ambient scribes could play a pivotal role if functionality, reliability, and physician trust can be further optimized.

## Introduction

Emergency physicians (EPs) are tasked with providing timely, quality patient care in a fast-paced care environment. In addition to direct patient care responsibilities, it is considered best practice for EPs to complete documentation in real time on shift for reasons including improved patient safety, documentation accuracy, medical-legal protection, and physician wellness. Because of this, a significant portion of the EP workday, upward of 40% of every shift, is consumed by administrative tasks such as the documentation of patient encounters in the electronic health record [1]. This administrative burden contributes to reduced job satisfaction and clinician burnout [2,3], as well as compromised patient interaction time [4]. This is particularly relevant in the field of emergency medicine (EM), as EPs have long been known to experience higher rates of burnout than physicians of any other specialty [5].

Progress in artificial intelligence (AI), particularly the development of large language models (LLMs) via deep neural networks, has created new opportunities to use existing natural language processing technology to generate comprehensive clinical documentation. These systems, such as the Nuance Dragon Ambient Experience (DAX) Copilot, typically use a smartphone- or computer-based microphone to capture and process physician-patient dialogues before transforming the information into structured clinical documentation. Some early studies in the outpatient setting have shown that AI-powered ambient scribe technology reduces time spent on notes with improved clinic visit experiences for physicians and patients [6,7], saving users of ambient scribes in one large medical group over 15,000 hours of physician time compared with nonusers [8]. Early pilot studies of ambient scribe technology have revealed large, statistically significant reductions in physician task load, burnout, and favorable utility [9], as well as a greater sense of engagement with patients during outpatient appointments [10].

However, other studies from the primary care setting suggest that ambient scribes do not make clinicians, as a group, more efficient [11]. Additionally, the accuracy of LLMs and ambient scribes has been questioned, as the programs have been found to commit errors of “hallucination,” where information not present in the input data is generated, and “omission,” where relevant information is excluded in the output, at rates that may be clinically significant [12]. One study of ambient scribe technology found 70% of generated notes contained errors, with an average of nearly 3 errors per note [13]. These concerns are shared by patients, with one study showing that while patients are generally open to the use of ambient scribes in their physicians’ offices, many have concerns about documentation accuracy and privacy [14]. As most studies of ambient scribe technology have been quantitative in nature, there is a relative lack of qualitative or mixed methods studies in the literature. Mixed methods approaches are particularly valuable in implementation-based quality improvement, where physician perspectives and usability directly influence successful adoption. Given that most of the available literature is quantitative, few existing studies examine the physician experience in their own words. Furthermore, most studies have taken place in the outpatient setting, with researchers calling for “further analyses...of AI scribes across various clinical scenarios and physician and patient demographic groups” [8].

The emergency department (ED) presents a significantly different environment from the outpatient setting due to patient acuity, clinical workflows, and environmental disruptions. Therefore, findings from the ambulatory setting may not generalize to the ED. Although there has been preliminary exploration of LLM utility in the ED for purposes other than note documentation [15,16], few studies have evaluated the use of ambient scribes in the ED setting. Due to the large and increasing volume of critical health care services delivered in the ED, there remains a pressing need to research the potential value of ambient scribe technology in this unique setting. Given the potential for significant improvement in workflow, documentation speed, and workplace satisfaction, we aimed to evaluate EPs’ satisfaction, preferences, perceived documentation efficiency, time spent documenting outside of shift, and trust in ambient scribe–generated notes. We additionally compared ambient scribing to in-person scribes and independent documentation and used qualitative thematic analysis to identify ED-specific challenges.

## Methods

### Inclusion and Exclusion Criteria

Inclusion criteria were (1) board certification in EM or pediatric EM and (2) ownership of an Apple iPhone device compatible with the ambient scribe app. All adult and pediatric EPs in the group were emailed an invitation and could opt in to being randomized to receive one of the available licenses. There were no exclusion criteria.

### Study Design and Objectives

This study used a cross-sectional, descriptive, nonexperimental mixed methods design. The objective of this pilot study was to evaluate the EPs’ satisfaction, preferences, perceived documentation efficiency, time spent documenting outside of shift, and trust in ambient scribe–generated notes compared with in-person scribes and independent documentation and to identify ED-specific usability challenges through thematic analysis of free-text responses. The primary independent variable was the documentation method (ambient scribe, in-person scribe, or independent documentation), and the dependent variables were physician-reported satisfaction, preferences, perceived documentation efficiency, time spent documenting outside of shift, trust in documentation accuracy, and perceived usefulness by note component. We performed a mixed methods analysis of quantitative and qualitative free-text responses from a cross-sectional survey of a pilot group of EPs to characterize their experiences with ambient scribe technology in the ED [17].

### Participant Characteristics, Sample Size, and Selection Procedures

Initially, 16 licenses for the software were obtained by the physician group. This is the maximum number of initial licenses that allocated funding allowed. All adult and pediatric EPs in the group were emailed an invitation to opt in to receive one of the available licenses. Among EPs who met the study’s inclusion criteria, 16 were selected to receive an initial license using a random number generator. As the license number was limited to 16 physicians as part of a pilot quality improvement project, a power analysis was not performed, as it was not feasible to adjust the number of pilot licenses. The physicians were selected among those who expressed an interest using an equally weighted randomizer. All physicians were board-certified in either EM or pediatric EM and practiced in one or more of 4 EDs within a single, urban medical system. Of the 16 physicians, 14 (87.5%) responded to the survey and represented a range of 0 to ≥21 years of practice: 0 to 5 years (n=3, 21.4%), 6 to 10 years (n=3, 21.4%), 11 to 15 years (n=2, 14.3%), 16 to 20 years (n=3, 21.4%), and ≥21 years (n=3, 21.4%).

### Setting and Study Dates

The individual sites included a large tertiary-care academic medical center, a midsize community hospital, a small community hospital, and a pediatric ED. All users attended an hour-long mandatory training session with a representative from the technology company, and all had the opportunity to participate in additional, optional training sessions. They were granted access to the ambient scribe on a rolling basis beginning September 1, 2024, and completed the survey between April 29, 2025, and May 27, 2025.

### Survey Instrument and Data Collection Procedures

An electronic survey was created by the research team and administered via REDCap (Research Electronic Data Capture) [18,19] and distributed by email to the group of EPs. The survey instrument was not adapted from a previously validated scale, and as such, no psychometric testing was performed. The survey functioned as the only instrument and consisted of multiple-choice and open-ended items. There was no masking, as the study used a voluntary, anonymous survey without treatment arms. As no inferential hypothesis testing was conducted, there are no inferential analytic strategies or data diagnostics to report. Quantitative data were summarized descriptively, and all responses were included without imputation.

Study data were collected and managed using REDCap electronic data capture tools hosted at Brown University Health.

The survey assessed physicians’ baseline use of in-person medical scribes and their experience with the ambient scribe and compared their preferences between in-person scribes, the ambient scribe, and independent documentation. Survey responses for multiple-choice questions were aggregated and presented as descriptive quantitative statistics without inferential analysis. 95% CIs were calculated using the Wilson score interval, which is validated for small sample sizes. There were no missing or imputed data.

### Thematic Analysis

A practical thematic analysis was performed by 2 members of the research team (TM and YB) on free-text responses in a 3-step process: reading the free-text responses, drafting effective codes or labels for concepts in the responses that were relevant to our study objective, and constructing meaningful themes in accordance with a framework as described by Saunders et al [20]. In step 1, we read the unedited free-text responses and independently wrote individual summary memos. In step 2, we performed data management and early data analysis by creating codes in the form of complete thoughts or sentences, rather than broad categories. We compared codes with other members of the team and developed a shared codebook. In step 3, we drafted themes independently before participating in a thematic analysis session in which we shared different perspectives and developed shared overarching, meaningful themes. The participating researchers were EPs with prior experience using the ambient scribe technology and employed by the same academic medical group as the participants. The themes were reported according to the frequency with which they arose in the free-text responses of survey participants. Saturation was not reached.

### Mixed Methods Integration

We relied on the results of both our quantitative and qualitative analyses to draw conclusions that provide a richer, more nuanced understanding of EPs’ experiences with ambient scribe technology than could be gathered from either analysis alone. We adhered to all SQUIRE 2.0 publication guidelines for quality improvement reporting [21].

The survey in its entirety is shown in Multimedia Appendix 1.

### Ethical Considerations

This human subject research study was deemed exempt by the local institutional review board (IRB; protocol 002825). The IRB approved a waiver of written informed consent. Participants received an email invitation containing IRB-approved information on the study’s purpose, procedures, risks, and benefits. The invitation text is available in Multimedia Appendix 1 with contact information redacted. All study data were deidentified. Participant data in the figures and appendix have been fully deidentified. A US $25 gift certificate was awarded to 2 study participants, selected at random among those who opted into the optional drawing.

## Results

Of the 16 EPs, 14 (87.5%) members of the cohort, consisting of 8 (57.1%) general EPs and 6 (42.9%) pediatric EPs, completed the survey during the study period. Among the cohort, 2 (12.5%) general EPs did not respond to the survey. Additionally, 8 (57.1%) respondents previously used in-person medical scribes in their routine practice and completed questions comparing their experience with the ambient scribe to in-person medical scribes, in addition to the standard questions about ambient scribe technology.

**Figure 1:**
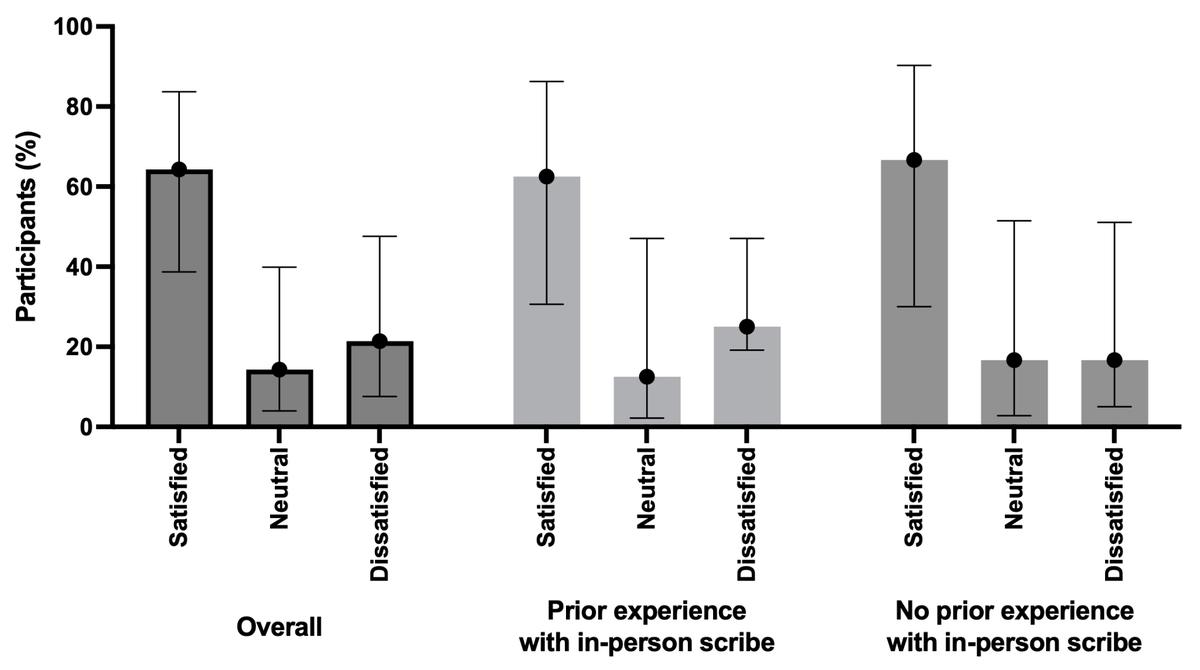
Overall satisfaction with artificial intelligence–powered ambient scribe technology among 14 board-certified emergency and pediatric emergency physicians who completed a cross-sectional survey across 4 emergency departments in a single urban health system. The satisfied category includes answers for very satisfied or satisfied. The dissatisfied category includes answers for very dissatisfied or dissatisfied.

Among 14 respondents, 9 (64.3%; 95% CI 38.7%-83.7%) reported feeling either satisfied or very satisfied with the ambient scribe, 2 (14.3%; 95% CI 4%-40%) felt neutral, and 3 (21.4%; 95% CI 7.6%-47.6%) reported feeling either dissatisfied or very dissatisfied with the ambient scribe (Figure 1). When asked about preferences between an in-person scribe, ambient scribe, or neither, 4 (28.5%; 95% CI 11.7%-54.6%) respondents preferred an in-person scribe, 7 (50%; 95% CI 26.8%-73.2%) preferred the ambient scribe, 3 (21.4%; 95% CI 7.6%-47.6%) were indifferent between the two, and none (95% CI 0%-20.6%) preferred independent documentation. Of the 8 respondents who reported using a medical scribe regularly before the introduction of the ambient scribe, 4 (50%; 95% CI 21.5%-78.5%) reported a preference for the ambient scribe.

**Figure 2:**
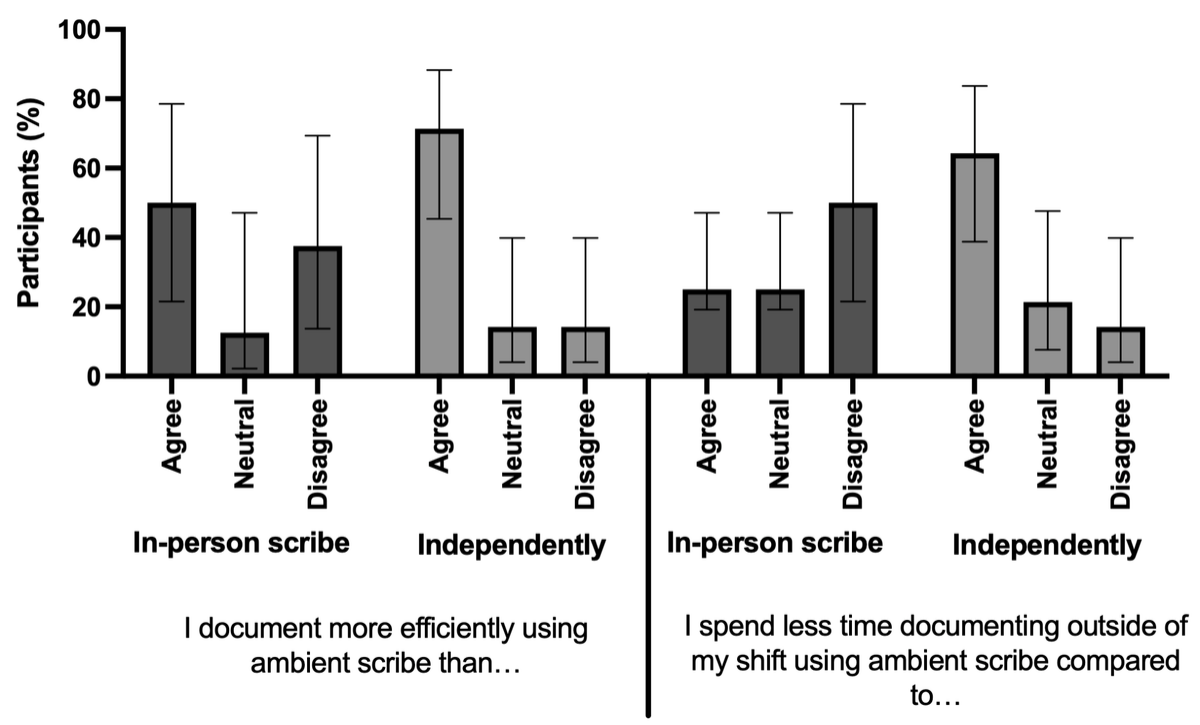
Physician-centered outcomes of documentation efficiency and time spent on documentation after shift using artificial intelligence–powered ambient scribe technology or an in-person scribe. Data were collected from 14 board-certified emergency and pediatric emergency physicians who completed a cross-sectional survey across 4 emergency departments in a single urban health system. The agree category includes answers for strongly agree or agree. The disagree category includes answers for strongly disagree or disagree.

In the series of questions specifically comparing the ambient scribe to independent documentation, 71.4% (10/14; 95% CI 45.4%-88.3%) of the respondents preferred the ambient scribe, 71.4% (10/14; 95% CI 45.4%-88.3%) reported improved efficiency with documentation, and 64.3% (9/14; 95% CI 38.7%-83.7%) reported decreased time spent outside of shift on documentation (Figure 2). When comparing an ambient scribe to an in-person scribe, 50% (4/8; 95% CI 21.5%-78.5%) of respondents reported improvement in efficiency with documentation, and 25% (2/8; 95% CI 19.2%-47.1%) reported decreased time spent outside of shift on documentation (Figure 2).

**Figure 3:**
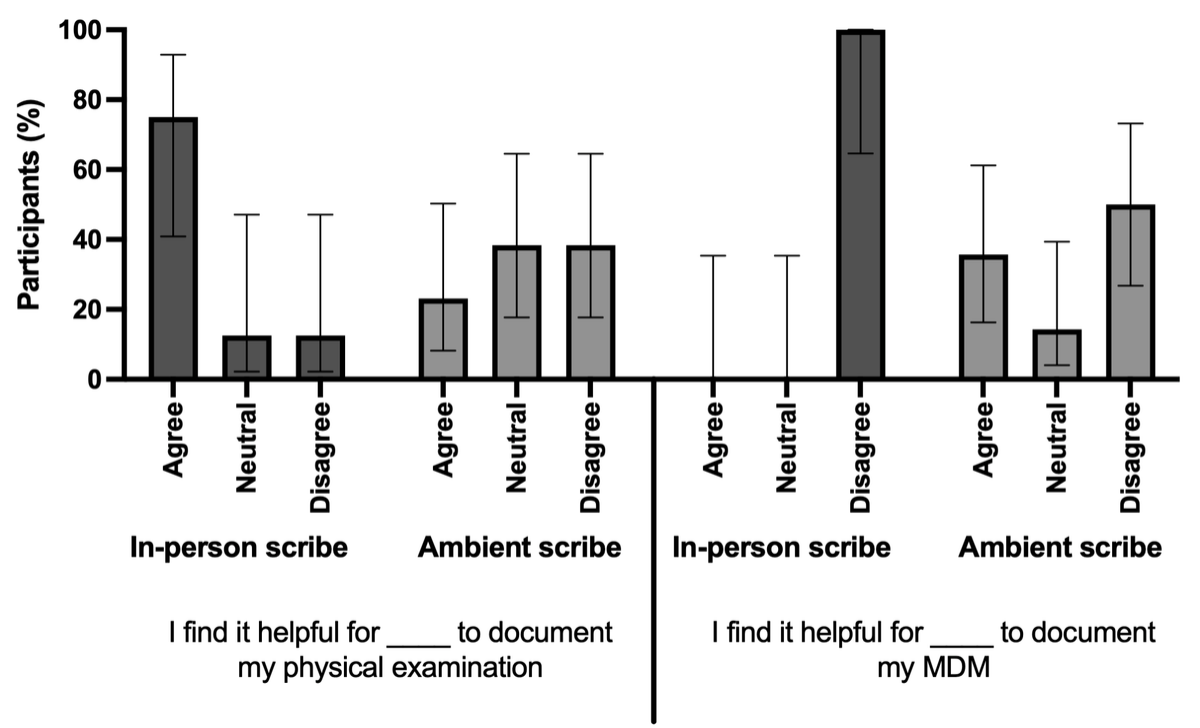
Preferences for the use of an ambient scribe or in-person scribes for elements of documentation are shown. Data were collected from 14 board-certified emergency and pediatric emergency physicians who completed a cross-sectional survey across 4 emergency departments in a single urban health system. The agree category includes answers for strongly agree or agree. The disagree category includes answers for strongly disagree or disagree. MDM: medical decision-making.

We asked respondents which documentation elements they found in ambient scribe technology to be most helpful. We found that only 23.1% (3/13; 95% CI 8.2%-50.3%) of the respondents reported the ambient scribe to be helpful for the physical examination (PE), and 35.7% (5/14; 95% CI 16.3%-61.2%) of the respondents reported the ambient scribe to be helpful for medical decision-making (MDM; Figure 3). Of the respondents who regularly use an in-person scribe, 75% (6/8; 95% CI 40.9%-92.9%) reported that they find it helpful to have scribes record the PE, and none (95% CI 0%-32.4%) reported it helpful to have scribes record their MDM. Overall, 42.9% (6/14; 95% CI 22.3%-69.2%) of respondents believe they can trust the ambient scribe’s documentation to be accurate, whereas 75% (6/8; 95% CI 40.9%-92.9%) of respondents with previous in-person scribe experience reported that they trust in-person scribes’ documentation to be accurate.

At the end of the multiple-choice questions, respondents were asked to “please leave any comments that may be helpful to understand your physician experience with DAX Copilot” in a free-text box. There were several qualitative comments shared by the survey respondents, as presented in Textbox 1. A thematic analysis of these open-ended responses found 5 broad, frequently cited themes, which included challenges due to the workplace environment, challenges due to patient population, workflow improvement, workflow harm, and narrow usefulness. These themes were pervasive in survey respondents’ free-text responses. As shown in Multimedia Appendix 2, the most common theme was workflow harm (6 instances), followed by workflow improvement (4 instances). The remaining themes were each mentioned 3 times.

**Free-text survey comments are presented verbatim without edits. These are the results of a cross-sectional survey that was conducted among 16 board-certified emergency and pediatric emergency physicians across 4 emergency departments within a single urban health system. Physicians were granted access to the ambient scribe and subsequently invited to complete an anonymous electronic survey.**

- “DAX copilot has been difficult to use in a busy ER setting given the noise in the department, elderly patient population, and nonverbal communication such as nodding.”
- “does a great job. there is a learning curve. you still need to review notes as they will need minor edits or corrections.”
- “I don’t have the option to use a scribe at work currently or recently (I think you asked about this at the beginning), so there is little point in asking me numerous questions about how DAX compares to a scribe. Unfortunately, only a minority of our ED patients can give a clear history, even when guided with straightforward ‘Yes’ or ‘No’ questions. Consequently, for the majority of ED patients, DAX generates a lousy history that then takes a lot of time to edit -- assuming you want a history that actually reflects the situation. Alternatively, if you don’t care much about what’s in the history (e.g. you are just interested in “filling in a blank” for billing and coding), then DAX is fine. DAX might work better just as a dictation tool -- instead of trying to listen in on a conversation and generate a history. For dictation, DAX seems to work better than Dragon which still -- after a decade -- still freezes up, drops words or parts of sentences. and at times makes egregious errors.”
- “Dax has been inaccurate, has weird wording and often takes me more time because I have to explain my use of it to patients to consent them and then heavily edit my notes. I do not trust Dax and have been very disappointed with is so far.”
- “DAX>Scribe when considering HPI gathering, though DAX is so verbose. The ideal state is to have Scribe tailor the HPI generated by DAX and Scribe tailor the physical. That is an expensive ideal state, so it is not practical. I find both to be very helpful. Scribes also grab patients' blankets and food. DAX cannot do that. Similarly, it is often nice on an overnight or other orphan shift to have a companion to shoot the breeze with. The morale boosting aspect of interacting with a young, eager (good) and appropriate scribe is another helpful aspect of scribe-physician interaction. Happy to answer any other questions.”
- “Dax copilot has completely changed my workflow when I see patients independently. I get my charts done real time and it doesn't slow down flow (like when I’d pile up 3-4 and do them in a period when I was waiting for results/studies to be done). It doesn’t get everything correct, but it’s as good/better than most scribes. If it was less clinic and more ED focused on its AI components, that would make it top notch!”
- “It is tough for physical exam and MDM, and it is not that helpful when patient is non-verbal or critically ill. Scribes are better for those patients, can get info from EMS/NH.”
- “I don’t think that DAX does a good job with the HPI or MDM. The HPI is disorganized. When I write/dictate an HPI, I organize it. DAX simply writes stream of consciousness of what happened during the encounter. The parent may talk about the location of the patient's pain, then mention that they missed school, then state that their older daughter had an appointment to go to, then come back to associated symptoms... and DAX will write it all in that haphazard order. It’s painful to read back! It’s like an awful medical student presentation! I have tried to change modes (to the more concise note), but I have the same issues. Similarly, the MDM uses my language, which is often diluted for patients (ex. ‘bellyache’).”
- “Only use for HPI documentation, often will redictate based on notes captured during hx taking. Does enhance ability to see multiple patients rapidly. Do not like MDM sections generated by DAX and never use. Has been some years since I used scribes- good scribes are far superior to DAX, but most scribes are not, and while some can be delegated to do other human tasks, ultimately slowed me down because of (enjoyable) human interaction/feedback/discussion outside of patient care.”
- “DAX copilot is efficient, accurate, and a huge job satisfier. The only concerns are it drains my personal cell phone battery and I don’t like bringing my personal cell phone into patient rooms.”

## Discussion

Most EPs surveyed reported a positive or neutral experience with ambient scribe technology, preferring the ambient scribe to independent documentation, citing improved efficiency and less time spent on documentation outside of shift in their responses to multiple-choice questions. Our thematic analysis yielded similar results but helped provide more granularity to physician experiences. Through this mixed methods approach, several important trends emerged. While respondents generally preferred the ambient scribe to documenting independently, they found its usefulness varied depending on the component of their required documentation for which it was being used. Many physicians identified the ambient scribe as useful for completing the history of present illness, while fewer found it helpful for PE or MDM. Trust in documentation accuracy was also limited, and lower than the level of trust reported for in-person scribes. Notably, unique challenges presented by the ED setting and its patient population were common themes, as respondents cited factors such as noise level and critically ill or nonverbal patients contributing to decreased satisfaction with ambient scribe technology.

This study is one of the first to show promise for the use of ambient scribe technology in the ED setting, complementing literature that calls for research on ambient scribes across additional clinical settings and physician groups [8]. Given that approximately half of all hospital-based health care in the United States is delivered in the ED, the setting represents an important, yet relatively understudied area in ambient scribe technology [22]. Most EPs in our study were overall satisfied with the technology and believed its use resulted in increased efficiency and time savings, consistent with studies that support its benefits in the outpatient setting [5-7]. One of these studies found that the implementation of ambient scribes by a large California medical group produced an estimated time savings of more than 15,700 hours for users compared with nonusers over a 1-year period [8]. Although our study was not designed to quantitatively assess time savings per EP or across EPs as a group, this remains an area for future research. To our knowledge, no research currently exists that evaluates the cost savings of the deployment of ambient scribe technology across a medical group or health system. This research would likely be consequential to health care administrators evaluating the advantages and risks of financial investment in the technology.

Ambient scribe technology has also recently shown promise in significantly reducing physician burnout in the ambulatory setting [23]. This is particularly relevant to the ED setting, as EPs have long been known to experience higher rates of burnout than any other group of physicians [5]. While our study did not directly evaluate the effect of ambient scribe technology on EP burnout, we consider it likely that less time spent documenting outside of shift would have a positive impact on reducing rates of EP burnout. Furthermore, ambient scribe technology has been shown to lead to a greater sense of engagement with patients during outpatient appointments [10]. While this outcome likely has a positive impact on reducing physician burnout, its effect on patients is likely positive, as well. A survey of patients at a clinic using ambient scribes in its practices showed that patients are generally open to the use of ambient scribes in their physicians’ offices and can see their potential benefits, although a significant number have concerns about documentation accuracy and privacy [14]. Interestingly, EPs surveyed in our study did not comment on experiencing a greater sense of patient engagement, but they shared patients’ concerns over documentation accuracy.

While our findings underscore the promise of ambient scribe technologies in alleviating documentation workload of EPs, several caveats exist. Importantly, EPs found that the AI-generated output still required significant editing, negatively impacting their views on its reliability and ability to improve their documentation efficiency, consistent with other studies showing that AI scribes remain error prone [12,13]. Importantly, the ambient scribe’s performance varied by note component, providing insight into an area that may be best suited for future improvement efforts. It is perhaps unsurprising that EPs in our study identified ambient scribes as most useful for documenting the history of present illness, as LLM-powered AI is widely considered useful for the summarization of information. Many electronic medical record systems have embedded capabilities to reduce the time spent on PE documentation through the use of templates. As such streamlining already exists, there may be less room for improved efficiency through the use of an ambient scribe or may require building on these already-existing solutions, such as integrating templated examinations with ambient scribing. Few other studies exist that examine physician experience and efficiency by note component, although this would be important to study in the future to identify potential gaps in current workflows where technology can improve efficiency.

Our pilot study has numerous limitations, the most significant of which is the small size of our population studied. Although most study participants responded to our survey, only 16 EPs across our hospital system were granted early access to the ambient scribe technology and were therefore eligible to participate in our study. While the 14 physicians who responded to our survey were well distributed in years of practice and EM vs pediatric EM training, their willingness to experiment with and adopt AI scribe technology may not be representative of all EM physicians, as they were self-selected and understood that they would be part of a group of early adopters of the technology at our institution. As there are multiple AI-powered ambient scribe technologies currently on the market, DAX Copilot may not be representative of the current state of ambient scribe technology due to product variation. Furthermore, we consider it likely that user experiences with ambient scribe technologies will change over time. There may be a “learning curve” while users adapt to the ambient scribe, and we expect aspects of the technology to evolve based on user preferences and feedback. Finally, our study did not evaluate patient experience or patient outcomes, which would be an important area of future research, as studies are currently limited.

This mixed methods pilot study is among the first to evaluate ambient scribe technology specifically in the ED, a unique environment that has been overlooked by the current body of literature focused on the outpatient setting. By combining quantitative survey outcomes with a qualitative thematic analysis of physician experiences, our study provides a more nuanced understanding of how this technology may function in a high-acuity and chaotic setting. Our findings highlight setting-specific challenges, such as noise and critical illness, variability in the trust of ambient scribe–generated output, and differences in the perceived utility of ambient scribing technology for different note components. By adding ED-specific evidence to the overall body of literature, our study offers guidance on how to tailor the technology for its use in the emergency setting. As the demand for efficiency in EM continues to grow in the face of persistent concerns over physician burnout and retention, scalable solutions such as AI scribes could play a pivotal role if their functionality, reliability, and physician trust can be further optimized. Areas of improvement may include improved functionality in noisy environments, decreasing errors and the need for manual editing, and development of enhanced or more tailored MDM sections. This technology may have particular value for institutions that do not have the resources to provide assistance with trained human scribes. The current value for sites with robust scribe programs is less clear. Future research should focus on longitudinal assessments of user experience and objective measures of documentation quality, time savings, and patient outcomes. With continued development and thoughtful integration, AI scribe technologies may ultimately help restore time for bedside patient care while improving physician well-being in the ED.

## Data Availability

The dataset for this investigation was submitted to JMIR with the manuscript and is available upon request by contacting the corresponding author (YB).

## Appendix

Multimedia Appendix 1 Full survey.

Multimedia Appendix 2 Results from a thematic analysis of free-text survey responses are shown. Five major themes were identified throughout the comments. The y-axis represents the number of times each theme was mentioned. Data were collected from 14 board-certified emergency and pediatric emergency physicians who completed a cross-sectional survey across 4 emergency departments in a single urban health system.

## Abbreviations

AI: artificial intelligence
ED: emergency department
EM: emergency medicine
EP: emergency physician
IRB: institutional review board
LLM: large language model
MDM: medical decision-making
PE: physical examination
REDCap: Research Electronic Data Capture
