# Supplementary material for: AI-Powered Ambient Scribe Technology Experiences Among Emergency Physicians: Cross-Sectional, Mixed Methods Pilot Survey Study
Source: JMIR Form Res. 2026 Mar 3;10:e80401. doi: 10.2196/80401 (PMC12996897; doi:10.2196/80401)
Supplement: Multimedia Appendix 1 [file formative_v10i1e80401_app1.pdf]

Physician Experience with DAX Copilot

Please complete the survey below. This is estimated to take 5-10 minutes total.

1) How many years have you been in practice as an attending physician?
- ◯ 0-5
- ◯ 6-10
- ◯ 11-15
- ◯ 16-20
- ◯ 21+

2) Are you a General EM physician or PEM-trained physician?
- ◯ General EM
- ◯ PEM-trained EM
- ◯ Both

3) Prior to the introduction of DAX Copilot, did you routinely use a scribe in your practice?
- ◯ Yes
- ◯ No

10%

Progress

The following questions compare your experience with in-person scribes relative to working independently with no scribe.

4) I prefer an in-person scribe to documenting independently.

- ◯ Strongly agree
- ◯ Agree
- ◯ Neutral
- ◯ Disagree
- ◯ Strongly Disagree
- ◯ N/A

5) I am more efficient documenting with an in-person scribe than independently.

- ◯ Strongly agree
- ◯ Agree
- ◯ Neutral
- ◯ Disagree
- ◯ Strongly Disagree
- ◯ N/A

6) In-person scribes improve my productivity with tasks outside of documentation while on shift (i.e., physical tasks).

- ◯ Strongly agree
- ◯ Agree
- ◯ Neutral
- ◯ Disagree
- ◯ Strongly Disagree
- ◯ N/A

7) I spend less time completing documentation outside of my shift when working with an in-person scribe relative to no scribe.

- ◯ Strongly agree
- ◯ Agree
- ◯ Neutral
- ◯ Disagree
- ◯ Strongly Disagree
- ◯ N/A

35%

Progress

The following questions compare your experience with DAX Copilot relative to working with an in-person scribe.

8) I prefer DAX Copilot to documenting independently.

- ◯ Strongly agree
- ◯ Agree
- ◯ Neutral
- ◯ Disagree
- ◯ Strongly Disagree
- ◯ N/A

9) I am more efficient documenting using DAX Copilot than documenting independently.

- ◯ Strongly agree
- ◯ Agree
- ◯ Neutral
- ◯ Disagree
- ◯ Strongly Disagree
- ◯ N/A

10) I spend less time completing documentation outside of my shift using DAX Copilot relative to no scribe.

- ◯ Strongly agree
- ◯ Agree
- ◯ Neutral
- ◯ Disagree
- ◯ Strongly Disagree
- ◯ N/A

50%

Progress

The following questions compare your experience with DAX Copilot relative to working with an in-person scribe.

11) I prefer DAX Copilot to working with an in-person scribe.

- ◯ Strongly agree
- ◯ Agree
- ◯ Neutral
- ◯ Disagree
- ◯ Strongly Disagree
- ◯ N/A

12) I am more efficient documenting using DAX Copilot than with an in-person scribe.

- ◯ Strongly agree
- ◯ Agree
- ◯ Neutral
- ◯ Disagree
- ◯ Strongly Disagree
- ◯ N/A

13) I spend less time completing documentation outside of my shift using DAX Copilot than working with an in-person scribe.

- ◯ Strongly agree
- ◯ Agree
- ◯ Neutral
- ◯ Disagree
- ◯ Strongly Disagree
- ◯ N/A

75%

Progress

The following questions help us understand your overall experience with in-person scribes and scribe technology.

14) I find it helpful for an in-person scribe to document my physical exam.
- ◯ Strongly agree
- ◯ Agree
- ◯ Neutral
- ◯ Disagree
- ◯ Strongly Disagree
- ◯ N/A

15) I find it helpful for DAX Copilot to document my physical exam.
- ◯ Strongly agree
- ◯ Agree
- ◯ Neutral
- ◯ Disagree
- ◯ Strongly Disagree
- ◯ N/A

16) I find it helpful for an in-person scribe to document my medical decision making.
- ◯ Strongly agree
- ◯ Agree
- ◯ Neutral
- ◯ Disagree
- ◯ Strongly Disagree
- ◯ N/A

17) I find it helpful for DAX Copilot to document my medical decision making.
- ◯ Strongly agree
- ◯ Agree
- ◯ Neutral
- ◯ Disagree
- ◯ Strongly Disagree
- ◯ N/A

18) I trust the documentation from an in-person scribe will be accurate.
- ◯ Strongly agree
- ◯ Agree
- ◯ Neutral
- ◯ Disagree
- ◯ Strongly Disagree
- ◯ N/A

19) I trust the documentation from DAX Copilot will be accurate.
- ◯ Strongly agree
- ◯ Agree
- ◯ Neutral
- ◯ Disagree
- ◯ Strongly Disagree
- ◯ N/A

20) I prefer the notes generated by DAX Copilot to those written by an in-person scribe.

- ◯ Strongly agree
- ◯ Agree
- ◯ Neutral
- ◯ Disagree
- ◯ Strongly Disagree
- ◯ N/A

100%

Progress

Please rank your overall efficiency with the following (1 = most efficient, 3 = least efficient):

		1	2	3
21)	In-person Scribe	◯	◯	◯
22)	DAX Copilot	◯	◯	◯
23)	Independent	◯	◯	◯

24) How satisfied are you with DAX Copilot?
- ◯ Very satisfied
- ◯ Satisfied
- ◯ Neutral
- ◯ Dissatisfied
- ◯ Very Dissatisfied
- ◯ N/A

25) If given the option of DAX Copilot, in-person scribe, or neither, I would choose:
- ◯ DAX Copilot
- ◯ Scribe
- ◯ Indifferent between DAX Copilot or in-person sc ribe
- ◯ Neither

26) Please leave any comments that may be helpful to understand your physician experience with DAX Copilot

____

If you would like to be entered into a random drawing to win a one of two $25 Visa gift cards, please enter your name and email below. This is entirely optional.

27) Name __________________________________

28) Email __________________________________
