# Supplementary figures and images for: AI-Powered Ambient Scribe Technology Experiences Among Emergency Physicians: Cross-Sectional, Mixed Methods Pilot Survey Study
Source: JMIR Form Res. 2026 Mar 3;10:e80401. doi: 10.2196/80401 (PMC12996897; doi:10.2196/80401)

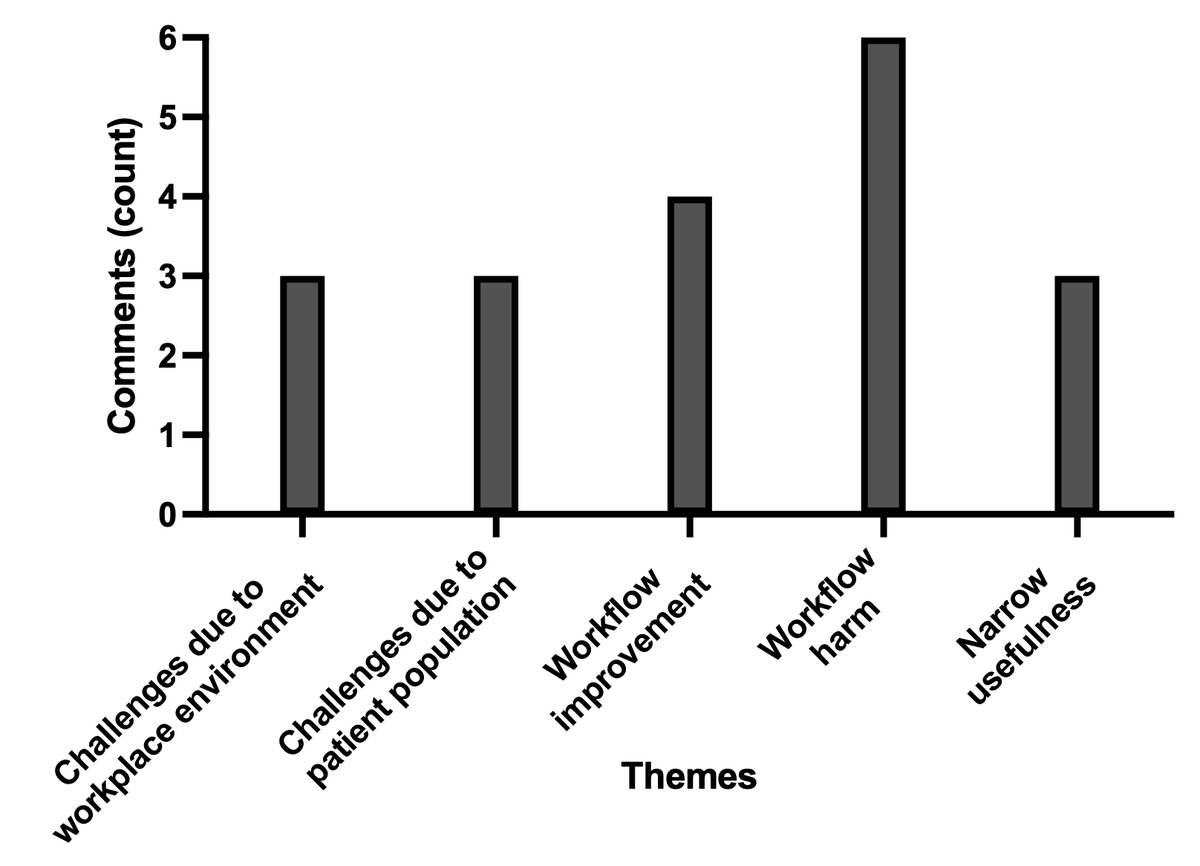

Supplement: Multimedia Appendix 2 [file formative_v10i1e80401_app2.png]
